# Superconductivity in a breathing kagome metals *R*Os_2_ (*R* = Sc, Y, Lu)

**DOI:** 10.1038/s41598-023-43621-w

**Published:** 2023-10-04

**Authors:** Karolina Górnicka, Michał J. Winiarski, Dorota I. Walicka, Tomasz Klimczuk

**Affiliations:** 1 Faculty of Applied Physics and Mathematics, Gdansk University of Technology, Ul. Narutowicza 11/12, 80-233 Gdańsk, Poland; 2 Advanced Materials Centre, Gdansk University of Technology, Ul. Narutowicza 11/12, 80-233 Gdańsk, Poland; 3 Department of Quantum Matter Physics, University of Geneva, 24 Quai Ernest-Ansermet, 1211 Geneva 4, Switzerland

**Keywords:** Superconducting properties and materials, Solid-state chemistry

## Abstract

We have successfully synthesized three osmium-based hexagonal Laves compounds *R*Os_2_ (*R* = Sc, Y, Lu), and discussed their physical properties. LeBail refinement of pXRD data confirms that all compounds crystallize in the hexagonal centrosymmetric MgZn_2_-type structure (*P6*_*3*_*/mmc*, No. 194). The refined lattice parameters are *a* = *b* = 5.1791(1) Å and *c* = 8.4841(2) Å for ScOs_2_, *a* = *b* = 5.2571(3) Å and *c* = 8.6613(2) Å for LuOs_2_ and *a* = *b* = 5.3067(6) Å and *c* = 8.7904(1) Å for YOs_2_. *R*Os_2_ Laves phases can be viewed as a stacking of kagome nets interleaved with triangular layers. Temperature-dependent magnetic susceptibility, resistivity and heat capacity measurements confirm bulk superconductivity at critical temperatures, *T*_*c*_, of 5.36, 4.55, and 3.47 K for ScOs_2_, YOs_2_, and LuOs_2_, respectively. We have shown that all investigated Laves compounds are weakly-coupled type-II superconductors. DFT calculations revealed that the band structure of *R*Os_2_ is intricate due to multiple interacting *d* orbitals of Os and *R*. Nonetheless, the kagome-derived bands maintain their overall shape, and the Fermi level crosses a number of bands that originate from the kagome flat bands, broadened by interlayer interaction. As a result, *R*Os_2_ can be classified as (breathing) kagome metal superconductors.

## Introduction

The kagome network, an edge-sharing triangular net, has first gained a significant research interest in the context of spin liquid phases^1–3^. An S = 1/2 ion placed on this 3-connected grid, with nearest-neighbor interactions present only, is an archetype of a geometrically frustrated magnetic system^4,5^.

**Figure 1:**
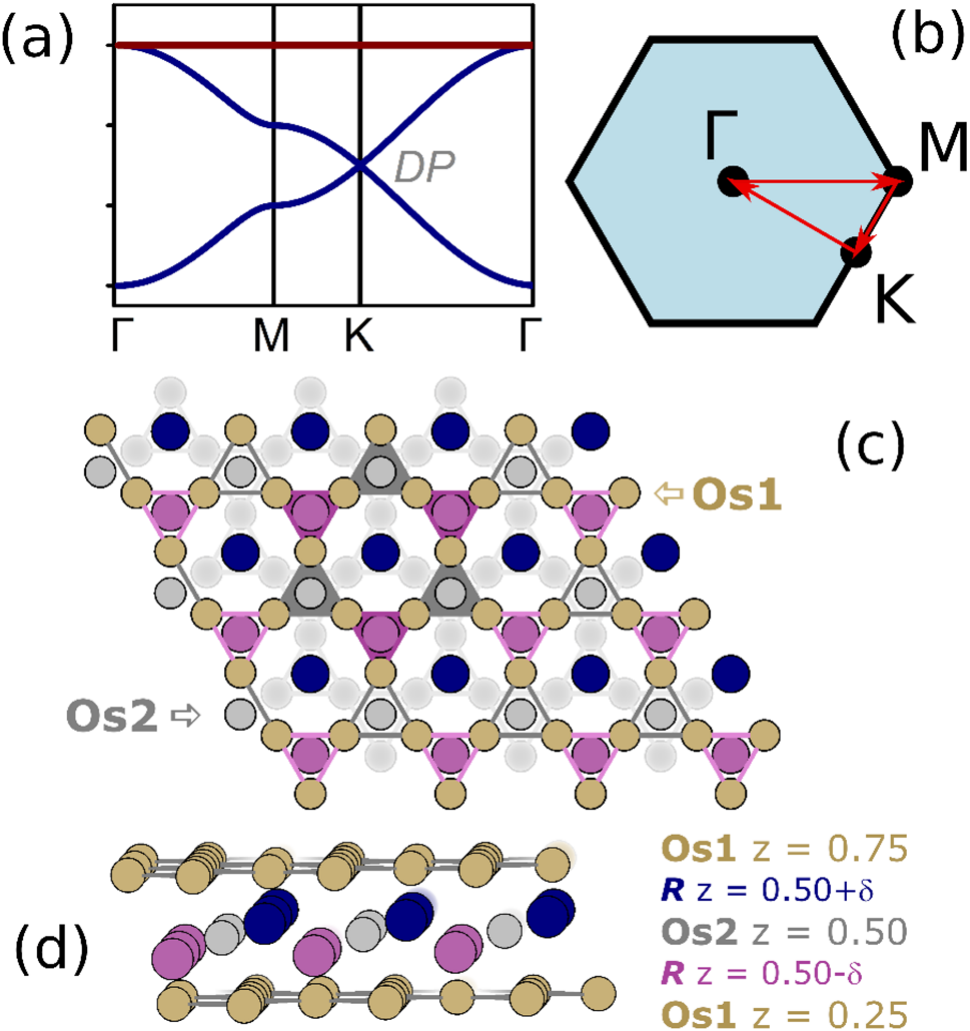
(**a**) 3-orbital tight-binding band structure of a kagome network with nearest-neighbor interactions only, showing a pair of Dirac bands crossing (DP) at the *K* point of the Brillouin zone. Locations of BZ points are shown schematically in (**b**). Crystal structure of *R*Os_2_ (**c**,**d**) shown as a stacking of Os1 breathing kagome layers separated by triangular planes of *R* and Os2. Note that purple and gray triangles highlighted in panel (**c**) are not equal in size, thus the symmetry of the 2D kagome network is reduced from p6m to p3m1.

Magnetic frustration is not, however, the only exotic physical behavior that is displayed by the kagome net. A 3-orbital tight binding model of the band structure of an isolated kagome consists of a pair of Dirac bands crossing at the *K* point of the Brillouin zone (BZ) and a flat band (see Fig. 1a,b). The density of states (DOS) for such a system consists of 3 van Hove singularities: one for the flat band and two associated with the Dirac band saddle point at *M*. The flat band results from the localization due to a destructive interference of electron wavefunction on the kagome lattice^6^. The presence of the flat band marks the instability of the system towards crystal lattice distortion or magnetism^7,8^. In real crystalline solid-state systems the flat band gains some dispersion due to next-nearest neighbor (NNN) interactions between the kagome atoms (either direct or via other atomic layers), but the DOS still displays a peak^9–11^, which is often associated with the occurrence of ferromagnetism or superconducting transition.

The hexagonal MgZn_2_-type structure, belonging to a large group of Laves intermetallic phases, can be seen as a stacking of kagome planes (2 per unit cell) separated by two triangular network^12^ (see Fig. 1c,d). In hexagonal Laves *R*Os_2_ (*R* = Sc, Y, Lu) the osmium Os1 atoms in the 6* h* Wyckoff position form a trigonally-distorted (breathing) kagome net in the *ab* plane, where two hexagons and two triangles meet at each net vertex. Two kagome layers per unit cell with *z* = 0.25 and 0.75 are stacked along the *c* direction, while shifted against each other and connected by Os2 atoms at the 2*a* site (see Fig. 1d). The close-packed *R*-Os2 layer is capped above and below by the kagome nets.

Recently, we succeeded in synthesizing three *R*Os_2_ (*R* = Sc, Y, Lu) hexagonal Laves compounds which were briefly reported in Ref.^13^ to be superconductors. Though the latter report was published almost 60 years ago, to the best of our knowledge, no detailed studies have been performed as yet, aimed at determining the superconducting and normal-state parameters of these compounds. In this manuscript we report the synthesis of polycrystalline samples of *R*Os_2_ (*R* = Sc, Y, Lu) and experimental characterization of their electronic properties by means of dc magnetization, electrical resistivity, and heat capacity measurements. The experimental data are supplemented by the results of electronic band structure calculations. Density functional theory (DFT) calculations reveal fairly complicated band structure of *R*Os_2_ due to multiple interacting *d* orbitals of Os and *R* metal. However, the overall shape of the kagome-derived bands is preserved, and the Fermi level is found within the region of high DOS that stems from the kagome flat bands that are broadened by interaction between the layers. *R*Os_2_ can be thus considered as the kagome metal superconductors.

## Experimental section

Polycrystalline compounds ScOs_2_, YOs_2_ and LuOs_2_ were prepared by using the standard arc-melting method. High-purity elements, i.e., (> 99.9 wt%, Onyxmet, Poland) were weighed in nominal stochiometric ratios and arc-melted together in an inert argon atmosphere on a water-cooled cooper hearth. A piece of zirconium was used as a getter material during the melting process. To improve chemical homogeneity, the ingots were remelted three times, flipping them over after each melting. Weight losses upon melting were negligible (< 0.5%). All samples were hard, silver in color and stable against air and moisture. Parts of each sample were wrapped in tantalum foil, sealed under vacuum in a quartz tube and annealed at 800 $^\circ{\rm C}$ for 7 days. The annealing temperature (800 $^\circ{\rm C}$) for all samples was chosen based on a known Y-Os phase diagram^14^. No melting was observed during the heating process.

Powder X-ray diffraction (pXRD) measurements of as-cast and annealed samples were carried out at room temperature using Bruker D2 Phaser diffractometer [Cu Kα radiation (λ = 1.5406 Å)] equipped with a LynxEye-XE detector. Full LeBail analysis of the obtained XRD patterns was performed using the Bruker DIFFRAC.SUITE TOPAS software. The magnetization measurements were performed using a Quantum Design Evercool II Physical Property Measurement System (PPMS) with a Vibrating Sample Magnetometer (VSM) function. Both zero-field-cooled (ZFC) and field-cooled (FC) data were collected from 1.7 to 7 K under an applied field of 10 Oe. The magnetization was also measured at various temperatures in the superconducting state (*T* < *T*_*c*_) as a function of the applied field. Magnetic measurements were performed on samples of arbitrary shape with a mass of about 15 mg. All thermodynamic and transport measurements were also performed in a PPMS system. Specific-heat measurements were carried out in zero field and field up to 3 T, using the two-τ time-relaxation method. Each sample was cut to a suitable size and mounted with the Apiezon N grease onto the α-Al_2_O_3_ measurement platform to ensure good thermal contact. Temperature- and magnetic-field-dependent electrical transport measurements were tested using a standard four-probe technique, in which Pt wires ($\varnothing =50\, \upmu\mathrm{m}$) were attached to the surface of polished samples by spot welding.

Electronic band structure and density of states calculations were performed by means of the density functional theory with the Perdew–Burke–Ernzerhof generalized gradient approximation (PBE GGA)^15^ of the exchange–correlation potential utilizing the ELK 5.2.14 all-electron full-potential linearized augmented plane wave + local orbitals (FP-LAPW + lo) code^16^. Crystal structures were taken from the Materials Project database^17^ (MP id: *mp-567612*, *mp-570670*, *mp-567590* for Sc-, Y-, and Lu-bearing compound, respectively) and were used without further relaxation. Calculations were conducted in the full- (with spin–orbit coupling) and scalar-relativistic (neglecting the SOC) on an 8 × 8 × 6 Monkhorst–Pack *k*-point mesh.

Tight binding models of kagome networks were created and solved using the Pybinding package^18^.

## Results

The room-temperature pXRD patterns of ScOs_2_, YOs_2_ and LuOs_2_ materials are presented in Fig. S1 in Supplementary Materials (SM). All compounds are reported to crystallize in a hexagonal centrosymmetric MgZn_2_-type structure (*P6*_*3*_*/mmc*, No. 194)^13^. The pXRD confirmed a good quality of all samples with a small amount of impurity phases (Os; *P6*_*3*_*/mmc*, No. 194). Annealing at 800 $^\circ{\rm C}$ does not effect on the XRD patterns, indicating that all compounds melt congruently. In a more detailed analysis of the data, the *P6*_*3*_*/mmc* phase was refined with the LeBail method. The LeBail fit to the powder diffraction pattern is represented by a black solid line (Fig. S1). The refined lattice parameters are *a* = *b* = 5.1791(1) Å and *c* = 8.4841(2) Å for ScOs_2_, *a* = *b* = 5.2571(3) Å and *c* = 8.6613(2) Å for LuOs_2_ and *a* = *b* = 5.3067(6) Å and *c* = 8.7904(1) Å for YOs_2_. All values are in very good agreement with the data reported previously^13^. Figure S2 in SM presents a schematic view of the hexagonal structure of *R*Os_2_. The 4*f* (1/3, 2/3, z) site is occupied by *R* atoms and the 2*a* (0,0,0) and 6*h* (x, 2x, 1/4) sites are occupied by Os atoms.

**Figure 2:**
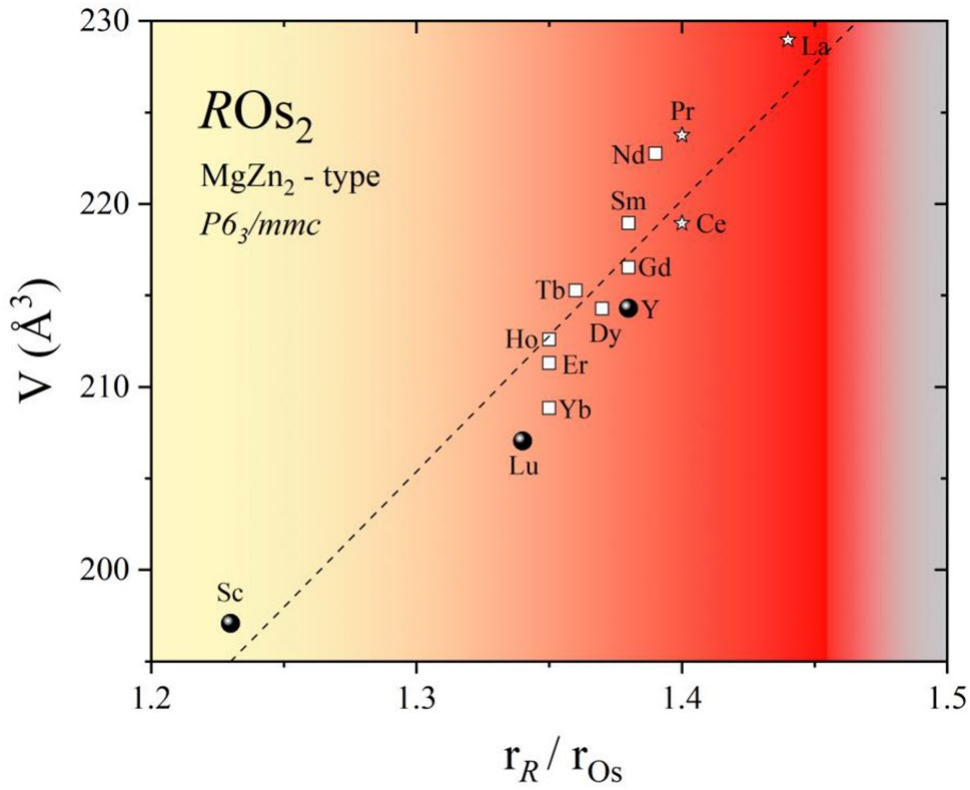
The unit cell volume vs the atomic radius ratio of the rare earth metal to osmium metal, r_R_/r_Os_. LaOs_2_, CeOs_2_, and PrOs_2_ form in the hexagonal phase under high pressure.

Figure 2 shows the unit cell volume (*V*) versus the atomic radius ratio of the rare earth metal to osmium metal (r_R_/r_Os_). The value of radii ratio is an important parameter governing the formation of the Laves phase structure, which ideally is 1.225^19,20^. The ratio of the known Laves phases often deviates from this ideal value (ranges from 1.05 to 1.70). The r_R_/r_Os_ ratio was calculated based on atomic radii values given by S.M. McLennan^21^. The unit cell volumes for the hexagonal structure of *R*Os_2_ (*R* = lanthanides from La to Yb) were taken from the ICSD database. As expected, the unit cell volume increases with an increase in the radius of the rare earth metal i.e., the smallest value is noted for ScOs_2_ and the largest for LaOs_2_. It is worthwhile to mention that for *R*Os_2_ compounds one can observe polymorphic transition point (PTP), where the crystal structure changes^22,23^. Typically, by conventional arc melting techniques, the C15 (cubic) phase is formed with the light *R*'s (La-Pr) and the C14 (hexagonal) phase with the heavier *R*'s (Pr-Lu). However, it has been observed that for LaOs_2_, CeOs_2_, and PrOs_2_ annealing under high pressure leads to the transformation of the cubic (MgCu_2_-type) to the hexagonal (MgZn_2_-type) crystal structure^24,25^. Moreover, M.S. Torikachvili reported^26^ that the transformation of CeOs_2_ can be accomplished at ambient pressure by annealing at temperatures between 500 and 950 °C.

**Figure 3:**
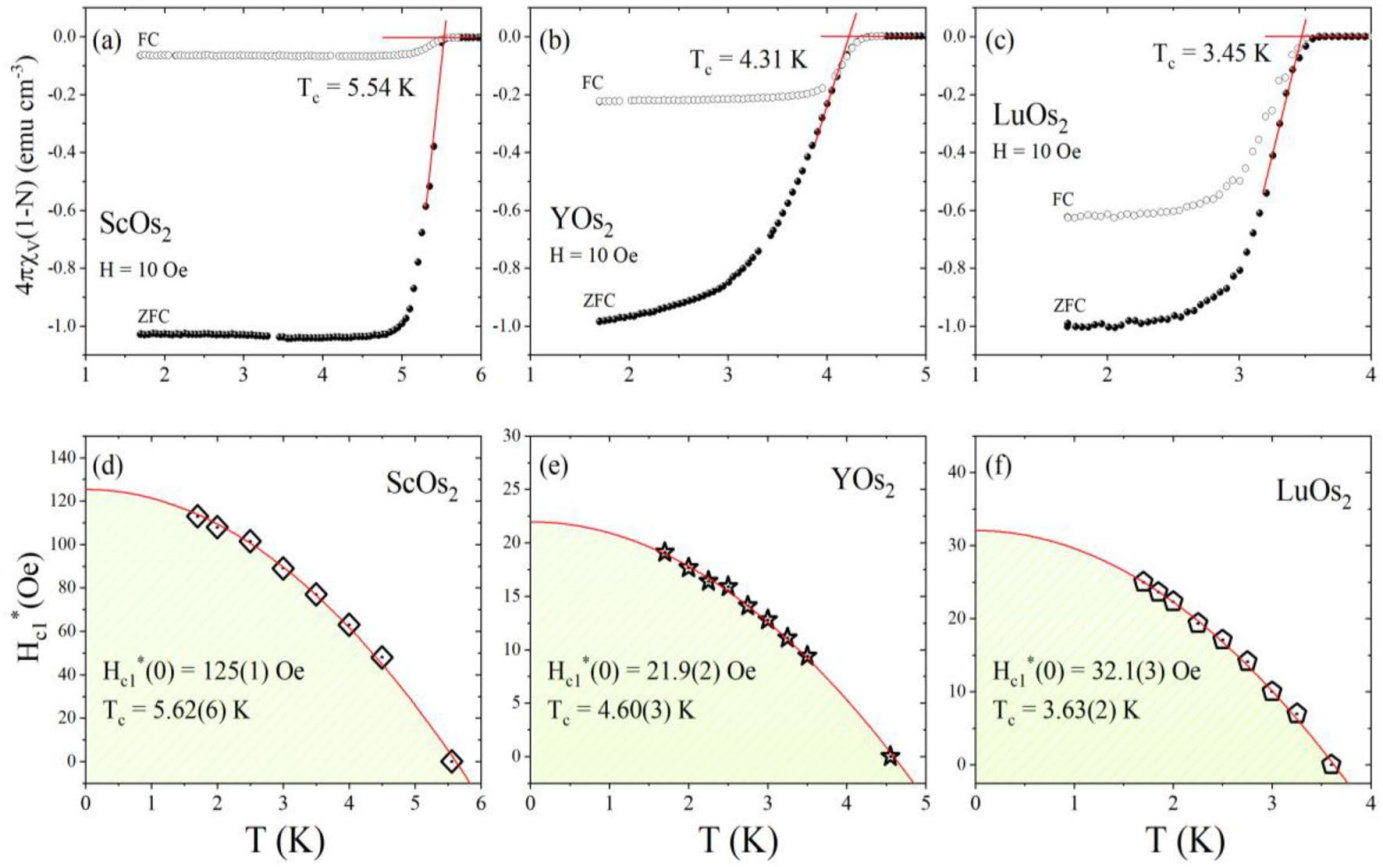
Zero-field-cooled (open circles) and field-cooled (full circles) temperature-dependent magnetic susceptibility data (*H* = 10 Oe) for ScOs_2_ (**a**), YOs_2_ (**b**), and LuOs_2_ (**c**). Temperature variation of the lower critical field for ScOs_2_ (**d**), YOs_2_ (**e**), and LuOs_2_ (**f**).

The superconducting properties of all compounds were first characterized by the measurement of temperature-dependent magnetization under zero-field-cooled and field-cooled conditions. Panels (a–c) of Fig. 3 present the volume magnetic susceptibility (defined as *χ* = *M*/*H* where *M* is the magnetization and *H* is the applied magnetic field) measured under an applied field of 10 Oe. The bifurcation of the ZFC and FC magnetic susceptibilities indicates the transition into the superconducting state. It can be seen that for the ZFC and FC signals, the transition is slightly broadened for all samples and reaches saturation at lower temperature. When corrected for the demagnetization effect, *N* = 0.33 for ScOs_2_ and LuOs_2_, and *N* = 0.73 for YOs_2_ (estimated from the *M*(*H*) data, discussed in SM), χ_ZFC_ approaches a value of − 1 at the lowest temperatures, indicating volume superconductivity. It should be noted that since the measured samples were in the form of individual chunks whose shape was not well defined, it is difficult to estimate the theoretically expected values of the *N*-factor. However, for the YOs_2_ sample, the rather large value of *N* and the absence of χ_ZFC_(*T*) saturation at the lowest temperatures may indicate that the superconducting Meissner fraction is not 100%. The diamagnetic signal of the FC measurement is weaker, likely caused by the flux line pinning, typically seen for polycrystalline samples of superconductors. The divergence of ZFC and FC signals is more pronounced for ScOs_2_ probably due to smaller grains and greater number of grain boundaries. The critical temperature was estimated as an intersection point between the extrapolated lines corresponding to the normal and superconducting state magnetic susceptibilities^27^. The *T*_*c*_ value is 5.54 K for ScOs_2_, 4.31 K for YOs_2_ and 3.45 K for LuOs_2_. It is worthwhile to mention that the values of critical temperature for compounds with Y and Lu agree well with the previous report^13^, while for ScOs_2_ the superconducting transition is observed at higher temperature than reported by V.B. Compton and B.T. Matthias (4.6 K^13^) or J.E. Hirsch (2 K^28^). The conservatively determined values thus obtained are larger than these reported for the cubic Laves phases with Ir e.g., ScIr_2_ (2.07 K^29^, 1.03 K^30^), YIr_2_ (2.18 K^29^), and LuIr_2_ (2.47 K^29^).

The field-dependent magnetization at different temperatures (*T* < *T*_*c*_) was measured to determine both the demagnetization factor, *N*, and estimate the value of the lower critical field, *H*_*c1*_(0). *M*(*H*) measured at selected temperatures for all compounds are depicted in Fig. S3 in SM. For all investigated samples the magnetization exhibits behavior observed for the conventional type-II superconductors^31–33^. The demagnetization factor was found assuming that the initial linear response to the field for an isotherm taken at *T* = 1.7 K is ideally diamagnetic. For an analysis of the lower critical field (${H}_{c1}^{*}$) the point corresponding to the first deviation from a linear response was estimated at each temperature. To precisely calculate this point, we followed the methodology described elsewhere^34–37^. In panels (d–f) of Fig. 3 the values of ${H}_{c1}^{*}$ are plotted as a function of temperature for all compounds. An additional point for *H* = 0 Oe is a zero-field transition temperature taken from the electrical resistivity measurement. The experimental ${H}_{c1}^{*}\left(T\right)$ data points were analyzed using the equation^38^:

1 $${H}_{c1}^{*}\left(T\right)= {H}_{c1}^{*}\left(0\right)\left[1-{\left(\frac{T}{{T}_{c}}\right)}^{2}\right]$$

where *T*_*c*_ is the superconducting critical temperature and ${H}_{c1}^{*}(0)$ is the lower critical field at 0 K. The solid red line through the data points shows a good agreement of the Ginzburg–Landau (GL) theory. Considering the demagnetization factor, the lower critical field, *H*_c1_(0) = *H*_c1_^*^(0)/(1 − *N*), at 0 K is calculated to be 187 Oe for ScOs_2_, 83 Oe for YOs_2_, and 48 Oe for LuOs_2_. It should be noted that since the demagnetization factor *N* for YOs_2_ is likely overestimated, the value of *H*_c1_(0) for this compound is likely smaller.

**Figure 4:**
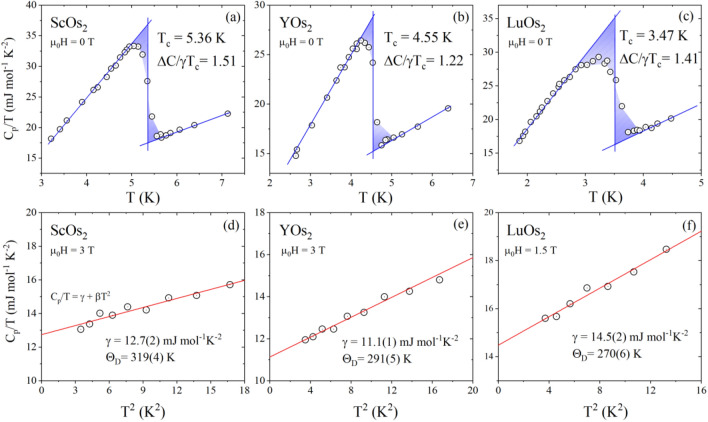
Temperature dependence of the zero-field specific heat in the vicinity of the superconducting phase transition for ScOs_2_ (**a**), YOs_2_ (**b**), and LuOs_2_ (**c**). *C*_*p*_/*T* vs *T*^*2*^ measured in a magnetic field: ScOs_2_ (**d**), YOs_2_ (**e**), and LuOs_2_ (**f**).

The results of low-temperature heat capacity (*C*_*p*_) measurements are summarized in Fig. 4. Panels (a–c) present the zero-field data plotted as *C*_*p*_/*T* versus temperature. The bulk nature of the superconductivity for all samples is confirmed by the pronounced heat-capacity jump on cooling through *T*_*c*_. To determine the critical temperature, we employed idealized equal entropy construction, which reflects the expected entropy balance between the normal state and the superconducting state at the superconducting phase transition. The *T*_*c*_’s equal 5.36 K, 4.55 K and 3.47 K for ScOs_2_, YOs_2_ and LuOs_2_, respectively, and agree with the magnetization data presented above.

**Figure 5:**
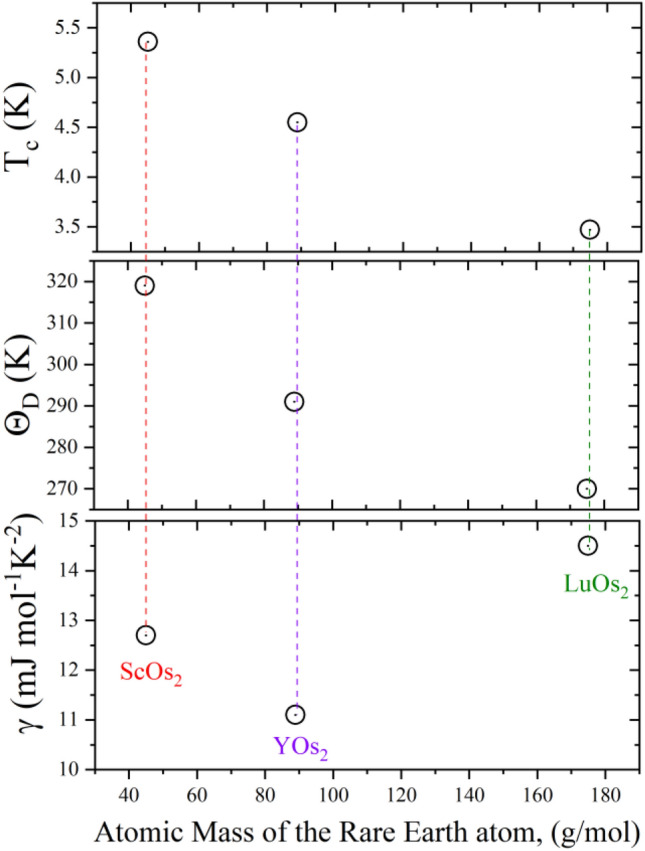
The critical temperature, the Debye temperature, and the Sommerfeld coefficient versus atomic mass of the rare earth atom in *R*Os_2_ compounds (*R* = Sc, Y and Lu).

Panels (d–f) of Fig. 4 depict the heat capacity data plotted as *C*_*p*_/*T* versus *T*^*2*^ under the magnetic field. In the normal state, the experimental data were fitted using the standard Debye expression *C*_*p*_/*T* = *γ* + *βT*^*2*^, where *βT*^*3*^ and *γT* are the phonon and electronic contribution to the specific heat, respectively. From the fit shown by the red solid line we obtained *γ* = 12.7(2) mJ mol^−1^ K^−2^, *β* = 0.18(2) mJ mol^−1^ K^−4^ for ScOs_2_, *γ* = 11.1(1) mJ mol^−1^ K^−2^, *β* = 0.24(1) mJ mol^−1^ K^−4^ for YOs_2_, and *γ* = 14.5(2) mJ mol^−1^ K^−2^, *β* = 0.29(2) mJ mol^−1^ K^−4^ for LuOs_2_. Using the Sommerfeld coefficient, the normalized specific heat jump, $\Delta$*C*/*γT*_*c*_, turned out to be 1.51, 1.22 and 1.41 for ScOs_2_, YOs_2_ and LuOs_2_, respectively. For all compounds, the estimated values are close to the BCS limit (1.43), suggesting a weakly-coupled superconductivity. In a simple Debye model, the *β* coefficient is related to the Debye temperature Θ_D_ through Θ_D_ $= {\left(\frac{12{\pi }^{4}}{5\beta }nR\right)}^{1/3}$, where *R* = 8.31 J mol^−1^ K^−1^ and n = 3. The resulting values of Θ_D_ are 319(4) K for ScOs_2_, 291(5) K for YOs_2_, and 270(6) K for LuOs_2_. Figure 5 shows obtained values of *T*_*c*_, Θ_D_, and *γ* versus atomic mass of the rare earth atom for investigated compounds.

The highest *T*_*c*_ is noticed for ScOs_2_ and may be related to the strengthening of the electron–phonon interaction. The highest and the lowest value of Θ_D_ is observed for ScOs_2_ and LuOs_2_, respectively, which can be qualitatively explained as the effect of the larger atomic weight of Lu comparing to Sc. The Sommerfeld coefficient, related to the density of states at the Fermi level, is slightly different for all compounds, with the largest value for LuOs_2_.

Having the estimated Debye temperature Θ_D_, and assuming that the Coulomb pseudopotential parameter µ* = 0.13^36,37,39–41^, we calculated the electron–phonon constant *λ*_e-p_ used the inverted McMillan’s formula^39^:

2 $${\lambda }_{e-p}= \frac{1.04 +{\mu }^{*}\mathrm{ln}\left(\frac{{\Theta }_{D}}{1.45{T}_{c}}\right)}{\left(1-0.62 {\mu }^{*}\right)\mathrm{ln}\left(\frac{{\Theta }_{D}}{1.45{T}_{c}}\right)- 1.04}$$

With these considerations, ${\lambda }_{e-p}$ is calculated to be 0.64, 0.63, and 0.59 for ScOs_2_, YOs_2_ and LuOs_2_, respectively. Determined values indicate weakly coupled superconductivity in all investigated compounds.

In addition, having the Sommerfeld coefficient and the electron–phonon coupling constant, the density of states at the Fermi energy *N*(E_F_) can be estimated:

3 $$N\left({E}_{F}\right)= \frac{3\gamma }{{\pi }^{2}{k}_{B}^{2}\left(1+{\lambda }_{e-p}\right)}$$

where k_B_ is the Boltzmann constant. *N*(E_F_) = 3.30 (ScOs_2_), 2.90 (YOs_2_) and 3.86 (LuOs_2_) states eV^−1^ per formula unit (f.u.).

**Figure 6:**
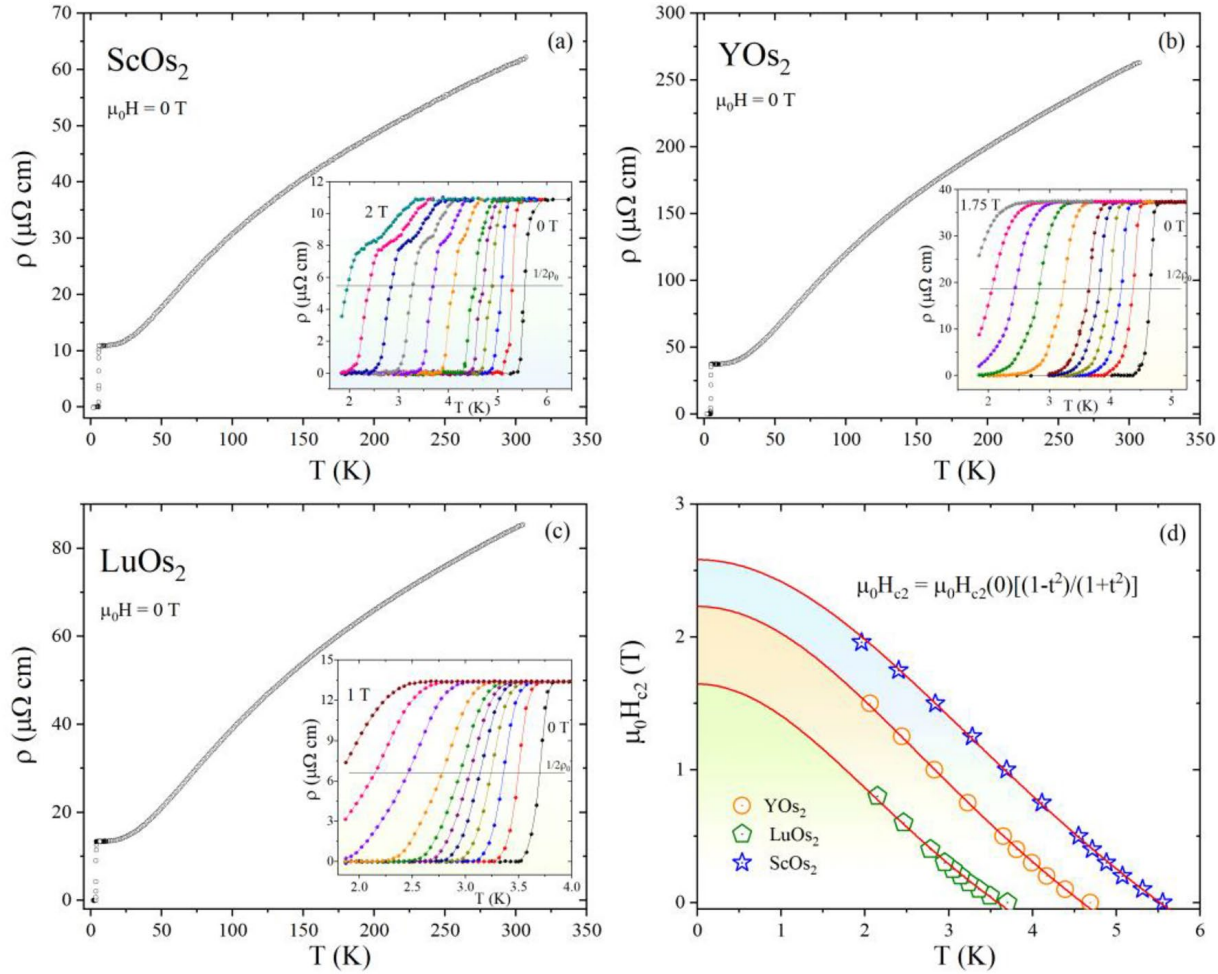
The electrical resistivity versus temperature measured in zero applied magnetic field for ScOs_2_ (**a**), YOs_2_ (**b**), and LuOs_2_ (**c**). Insets show the superconducting transition under various magnetic fields. (**d**) The temperature dependence of the upper critical field of all compounds, determined from electrical resistivity measurements.

The results of electrical resistivity measurements for all samples are summarized in Fig. 6a–c. Resistivity shows metallic behavior for all studied compounds with a residual resistivity ratio (RRR = ρ(300)/ρ(7)) of 5.7 for ScOs_2_, 7.1 for YOs_2_, and 6.4 for LuOs_2_, which is either attributable to the sample’s polycrystalline nature and grain boundaries or intrinsic. One can observe that the resistivity drops abruptly to zero, confirming that a superconducting transition occurs in all investigated compounds, which was also confirmed by a large diamagnetic signal and a significant specific heat jump at *T*_c_. To obtain the upper critical field (see insets of Fig. 6a–c), *H*_*c2*_(*T*), we measured the resistivity at various magnetic fields (*μ*_*0*_*H* = 0, 0.1, 0.2, 0.3, 0.4, 0.5, 0.75, 1, 1.25, 1.5, 1.75, and 2 T for ScOs_2_; *μ*_*0*_*H* = 0, 0.1, 0.2, 0.3, 0.4, 0.5, 0.75, 1, 1.25, 1.5, and 1.75 T for YOs_2_, and *μ*_*0*_*H* = 0, 0.05, 0.1, 0.15, 0.2, 0.25, 0.3, 0.4, 0.6, 0.8, and 1 T for LuOs_2_). As expected, with increasing magnetic field, the superconducting transition shifts to lower temperature. For ScOs_2_, a two-step transition is seen when the magnetic field is applied. The origin of this behavior is unknown, although, it might originate from the surface or filamentary superconductivity, with a higher critical field. For all investigated compounds, the upper critical field (*μ*_*0*_*H*_*c2*_(0)) is determined by the temperature when the resistivity drops to 50% of the normal-state value and is plotted as a function of temperature in the Fig. 6d. According to the Ginzburg–Landau (GL) theory, the *μ*_*0*_*H*_*c2*_ value at 0 K can be estimated using the expression^42–44^:

4 $${\mu }_{0}{H}_{c2}\left(T\right)={{\mu }_{0}H}_{c2}\left(0\right)\frac{\left(1-{t}^{2}\right)}{\left(1+{t}^{2}\right)}$$

where t = *T*/*T*_*c*_ and *T*_*c*_ is a fitting parameter (transition temperature at zero magnetic field). The fitting line from the GL relation fairly well describes the experimental data for all compounds and one can obtain the values of *μ*_*0*_*H*_*c2*_(0): 2.58(1) T for ScOs_2_, 2.23(2) T for YOs_2_, and 1.64(5) T for LuOs_2_. The paramagnetic limiting field (*μ*_*0*_*H*_*P*_) is given by *μ*_*0*_*H*_*P*_ = ${\Delta }_{0}/\sqrt{2}{\mu }_{B}$ (${\Delta }_{0}$ is the zero-temperature superconducting gap, and ${\mu }_{\mathrm{B}}$ is the Bohr magneton), which can be expressed as *μ*_*0*_*H*_*P*_ = 1.86 *T*_*c*_, yielding *μ*_*0*_*H*_*P*_ ~ 9.9, 8.5, and 6.5 T for ScOs_2_, YOs_2_ and LuOs_2_, respectively. In all cases the experimental values of *µ*_*0*_*H*_*c2*_(0) are much smaller than the Pauli limiting field, suggesting that all compounds are the conventional type-II superconductors. Table S1 (SM) gathers *μ*_*0*_*H*_*c2*_(0) values obtained from GL and WHH models.

Consequently, the coherence length, ξ_GL_, can be estimated using the Ginzburg–Landau formula $H_{{c2}} = \Phi _{0} /2\pi \xi _{{GL}}^{2}$, where Ф_0_ = hc/2e is the quantum flux. Having *μ*_*0*_*H*_*c2*_(0) values, the GL coherence length ξ_GL_ is 113 Å for ScOs_2_, 121 Å for YOs_2_, and 141 Å for LuOs_2_. The superconducting penetration depth λ_GL_(0) can then be obtained from *H*_*c1*_(0) and ξ_GL_(0) using the relation: ${H}_{c1}= \frac{{\Phi}_{0}}{4\pi {\lambda }_{GL}^{2}}\mathrm{ln}\frac{{\lambda }_{GL}}{{\xi }_{GL}}.$ The value is found to be *λ*_GL_(0) = 1513 Å for Sc variant, *λ*_GL_(0) = 2440 Å for Y variant, and *λ*_GL_(0) = 3270 Å for Lu variant. The obtained values are comparable with these reported for the cubic Laves phases e.g., BaIr_2_ (1520 Å^45^), SrIr_2_ (2370 Å^36^) or SrRh_2_ (2291 Å^36^). Furthermore, the GL parameter ${\upkappa }_{GL}$= λ_GL_/ξ_GL_ equals 13, 20, and 23 for ScOs_2_, YOs_2_ and LuOs_2_, respectively, which are clearly larger than 1/$\sqrt{2}$, implying that all investigated Laves compounds are the type-II superconductors. Finally, the thermodynamic critical field can be obtained from *κ*_GL_, *H*_*c1*_, and *H*_*c2*_ using the formula: ${H}_{c1}{H}_{c2}={H}_{c}^{2}\mathrm{ln}{\upkappa}_{GL}$, yielding *µ*_*0*_*H*_*c*_ = 136 mT for ScOs_2_, 78 mT for YOs_2_, and 50 mT for LuOs_2_. The superconducting and normal state parameters of all compounds are gathered in Table 1.

**Table 1:** Superconducting and normal state parameters of *R*Os_2_ (*R* = Sc, Y, Lu).

Parameter	Unit	ScOs_2_	YOs_2_	LuOs_2_
*T*_*c*_	K	5.36	4.55	3.47
*µ*_*0*_*H*_*c2*_(0)	T	2.58	2.23	1.64
*µ*_*0*_*H*_*c1*_(0)	mT	18.7	8.3	4.8
*µ*_*0*_*H*_*c*_	mT	136	78	50
λ_e-p_	–	0.64	0.63	0.59
ξ_GL_ (0)	Å	113	121	141
λ_GL_ (0)	Å	1513	2440	3270
κ_GL_	–	13	20	23
*ℽ*	mJ mol^−1^ K^−2^	12.7(2)	11.1(1)	14.5(2)
Θ_D_	K	319(4)	291(5)	270(6)
RRR	–	5.7	7.1	6.4
Δ*C*_*p*_/*ℽT*_*c*_	–	1.51	1.22	1.41
*N*(E_F_)	eV^−1^ f.u.^−1^	3.30	2.90	3.86

**Figure 7:**
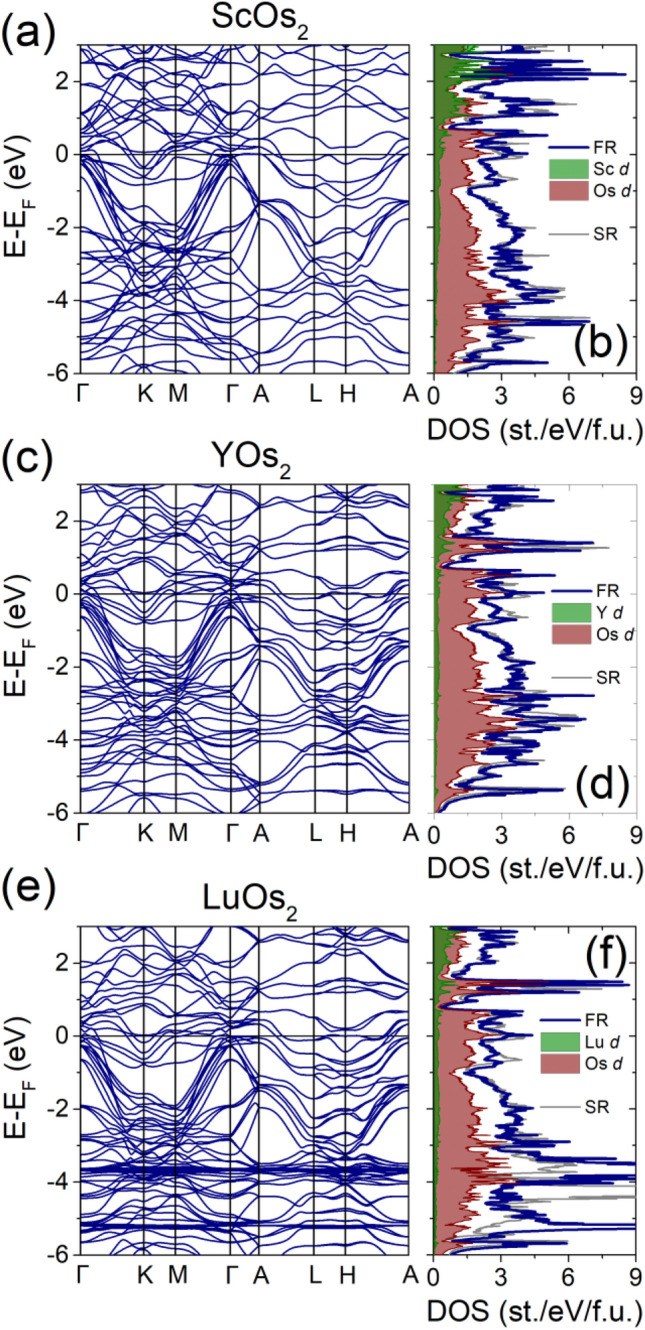
Band structure and electronic density of states for ScOs_2_ (**a**,**b**), YOs_2_ (**c**,**d**), and LuOs_2_ (**e**,**f**). In all three compounds the DOS(E_F_) is dominated by the contribution of Os 5*d* states. Besides the splitting of the completely occupied 4*f* band in LuOs_2_ (peak ca. − 4 to − 6 eV below the E_F_), the difference between fully- (FR; blue line in panels **b**,**d**,**f**) and scalar-relativistic (SR; gray line) is rather small.

To further understand the electronic structure of ScOs_2_, YOs_2_ and LuOs_2_ we performed electronic DOS and band-structure calculations (see Fig. 7). DFT calculations show that the DOS in the vicinity of the Fermi level in all three cases is dominated by Os 5*d* states, with electropositive elements (Sc, Y, Lu) acting mostly as electron donors. Inclusion of the spin–orbit coupling does not significantly affect the DOS(E_F_).

**Figure 8:**
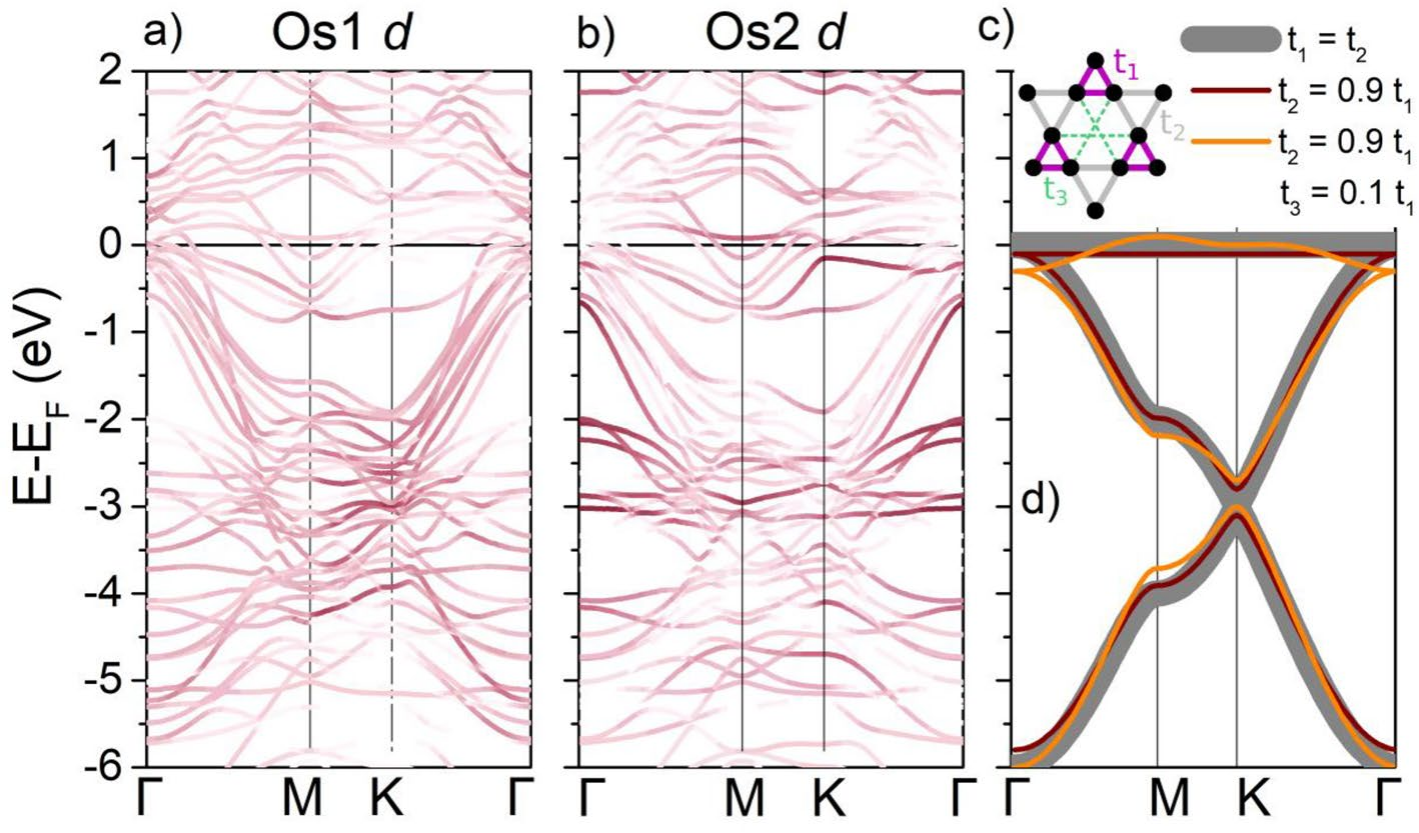
Band structure of ScOs_2_ with Os1 *d* (**a**) and Os2 *d* (**b**) contribution highlighted (proportional to the color intensity). Os1 dominates the kagome-like bands between 0 and − 6 eV, while Os2 contributed mostly to a number of weakly dispersive bands between − 2 and − 3 eV. Panel (**d**) shows the tight binding band structure of a kagome system within 3 approximations: in the simplest case (thick gray lines) only nearest-neighbor interactions are considered and all the nearest neighbor tight binding hopping integrals are set to be equal (t_1,_ t_2_ = − 1), resulting in a perfect p6m kagome. When the hexagonal symmetry is broken in breathing kagome (brown line; t_1_ = − 1, t_2_ = 0.9), the Dirac point at *K* is gapped, but the flat band remains intact. Inclusion of next-nearest neighbor interaction (orange line, t_1_ = − 1, t_2_ = 0.9, t_3_ = 0.1) results in the flat band attaining some dispersion. The three tight-binding models are schematically drawn in panel (**c**).

The broad peak-like feature of DOS within 1 eV around the Fermi level stems from several weakly dispersive Os *d*-dominated bands, followed by a number of highly dispersive bands between ca. 0 and − 6 eV (Fig. 8a,b). This is highly reminiscent of a generic tight-binding kagome band structure, as shown in Fig. 8c,d. Kagome-like bands bear a strong contribution of Os1 *d* states, while the Os2 *d* mostly contributes to a set of weakly dispersive bands around − 2 to − 3 eV below the E_F_.

Obviously, the band structure is much more complicated than e.g. in *A*V_3_Sb_5_ kagome superconductors^46^ or in CoSn^10^. This is due to: (1) the presence of two Os layers (kagome + trigonal) that are within the interacting range, resulting in significant hybridization of kagome bands, (2) fairly large unit cell consisting of 2 individual kagome planes, (3) trigonal distortion (breathing kagome), lifting the degeneracy at the *K* point and resulting in a gap between the Dirac bands.

Nevertheless, the set of weakly dispersive bands forming the DOS around the Fermi level can be traced back to the kagome flat band that is “bent” by interactions (Fig. 8c).

## Summary

In summary, a detailed investigation of superconducting and normal state properties of *R*Os_2_ (*R* = Sc, Y, Lu) hexagonal Laves compounds is presented. Magnetic susceptibility, electrical resistivity, and specific heat capacity measurements showed that *R*Os_2_ (*R* = Sc, Y, Lu) are type-II superconductors with transition temperatures of *T*_*c*_ = 5.36, 4.55, and 3.47 K, respectively. For ScOs_2_ the superconducting transition is observed at a higher temperature than reported previously, but the lack of details in the previous reports does not allow us to speculate about why the *T*_*c*_ is different from ours. The normalized specific heat jumps, Δ*C*/*γT*_*c*_, is calculated to be 1.51, 1.22, and 1.41 for ScOs_2_, YOs_2_, and LuOs_2_, evidencing the bulk nature of the superconductivity in these materials. Our band structure calculations showed that the dominating contribution to DOS(E_F_) came from 5*d* states of Os atoms. The overall domination of Osmium 5*d* states suggests that *R*Os_2_ compounds are Os 5*d*-band metals and that 5*d* electrons play the dominant role in superconductivity.

In addition to the superconducting properties, the studied compounds also exhibit a unique lattice structure. The Os atoms in these compounds form a trigonal breathing kagome lattice, a distorted variant of the hexagonal kagome. The band structure of the *R*Os_2_, although complicated, can be traced back to a generic kagome band model, modified by the breathing distortion and interlayer interactions.

### Supplementary Information

Supplementary Information.

## Data Availability

The datasets generated during and/or analysed during the current study are available from the corresponding author on reasonable request.
