# Supplementary material for: Superconductivity in a breathing kagome metals ROs2 (R = Sc, Y, Lu)
Source: Sci Rep. 2023 Oct 4;13:16704. doi: 10.1038/s41598-023-43621-w (PMC10550963; doi:10.1038/s41598-023-43621-w)
Supplement: Supplementary file 1 — Supplementary Information. [file 41598_2023_43621_MOESM1_ESM.docx]

**Supplementary Materials**

**Superconductivity in a breathing kagome metals *R*Os_2_ (*R* = Sc, Y, Lu)**

Karolina Górnicka^1,2,3^, Michał J. Winiarski^1,2^, Dorota I. Walicka^3^ and Tomasz Klimczuk^1,2^

*^1^ Faculty of Applied Physics and Mathematics, Gdansk University of Technology,*

*ul. Narutowicza 11/12, 80-233 Gdańsk, Poland,*

*^2^Advanced Materials Centre, Gdansk University of Technology,*

*ul. Narutowicza 11/12, 80-233 Gdańsk, Poland,*

*^3^ Department of Quantum Matter Physics, University of Geneva,*

*24 Quai Ernest‑Ansermet, 1211 Geneva 4, Switzerland.*

**Powder x-ray diffraction**

**Figure S1** presents the powder x-ray diffraction pattern for ScOs_2_, YOs_2_ and LuOs_2_ together with the LeBail refinement. All compounds crystallize in hexagonal centrosymmetric MgZn_2_ – type structure (*P6_3_/mmc*, No. 194). A small amount of impurity phase (Os; space group *P6_3_/mmc*, No. 194 (JCPDS card PDF 00-006-0662)) is observed for ScOs_2_ and YOs_2_.


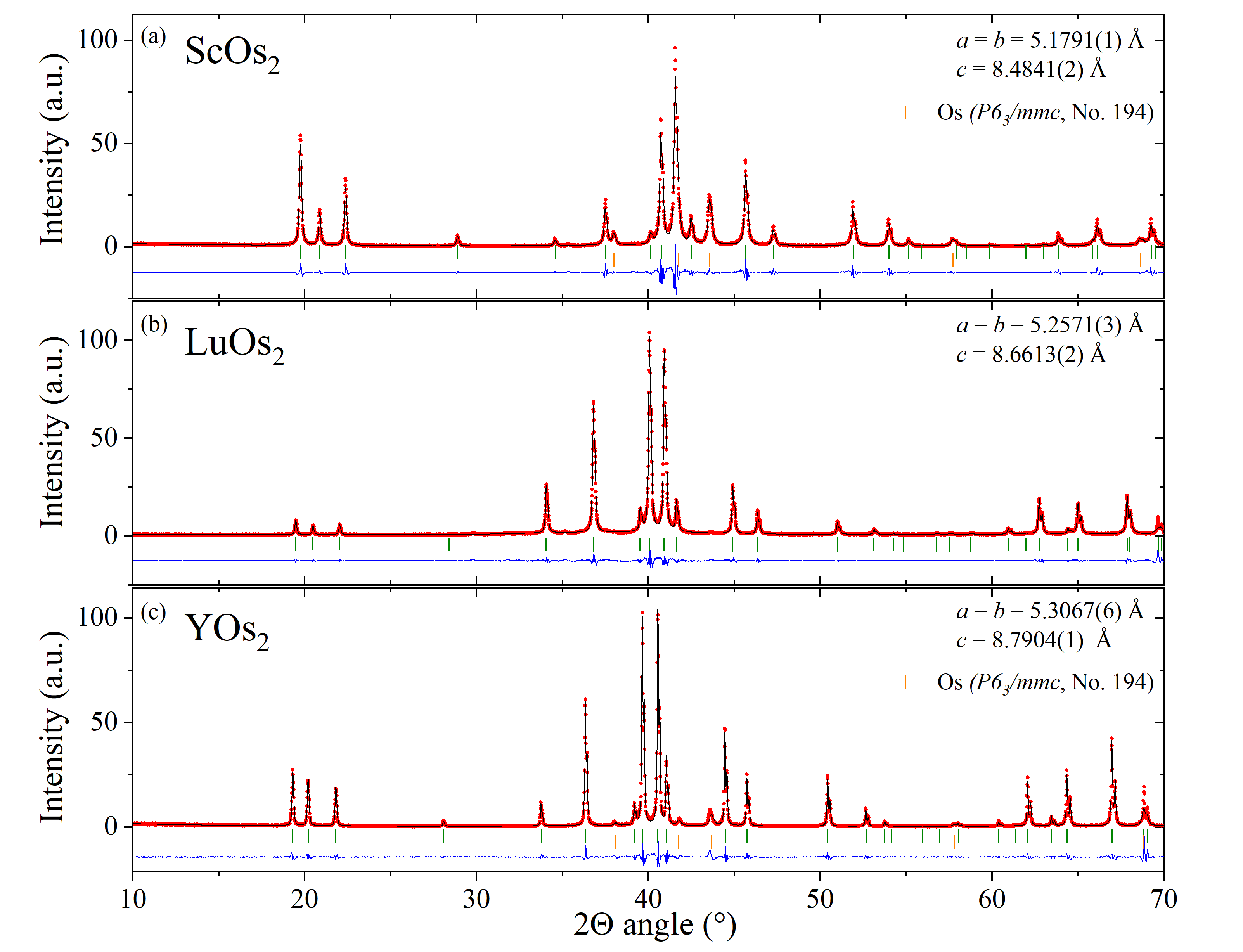


**Figure S1.** Powder x-ray diffraction pattern (pXRD) (red points) together with the LeBail refinement profile (black solid line) for *R*Os_2_ (*R*=Sc, Lu and Y) compounds.

**Figure S2** presents a schematic view of the hexagonal structure of *R*Os_2_. The 4*f* (1/3, 2/3, z) site is occupied by RE atoms and the 2*a* (0,0,0) and 6*h* (x, 2x, 1/4) sites are occupied by Os atoms.


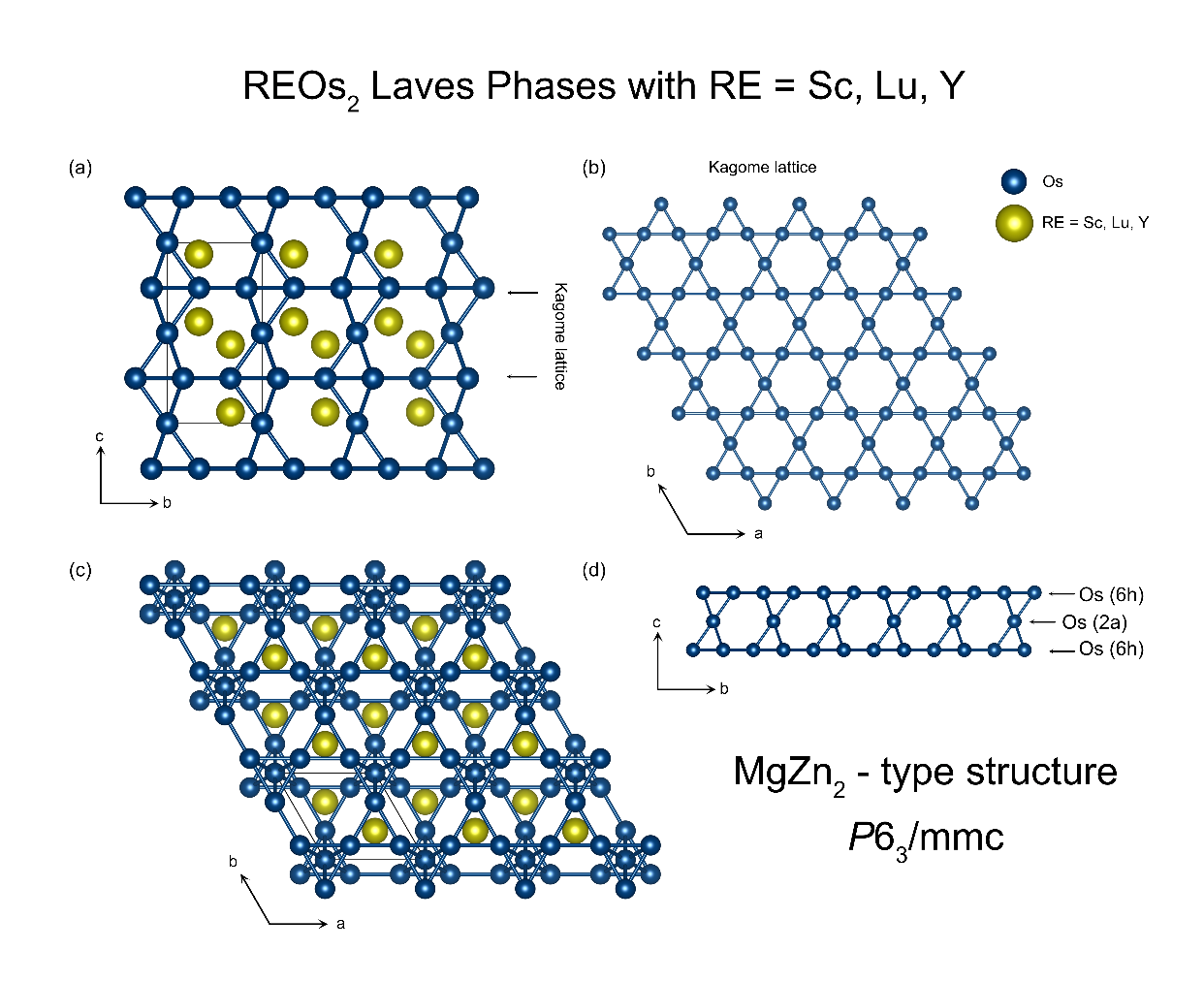


**Figure S2**. Crystal structure of *R*Os_2_ viewed along different orientations with *R* and

Os atoms represented by yellow and blue balls, respectively.

**Magnetic property measurements**

**Figure S3** depicts the magnetization as a function of magnetic field at several, selected temperatures below the superconducting transition temperature *T*_c_ for all studied compounds. For each temperature, the experimental data obtained in small magnetic fields were fitted using the proportionality *M_fi_*_t_ = −*aH*, appropriate for a full Meissner state. Comparing the value of prefactor *a* derived from the isotherm taken at *T* = 1.7 K with the ideal diamagnetism quantified as −1/4π, the demagnetization factor *N* = 0.33 for ScOs_2_ and LuOs_2_ and N = 0.73 for YOs_2_ was found. These values are comparable to those expected for a cylinder at different height-to-radius ratios ^1^.


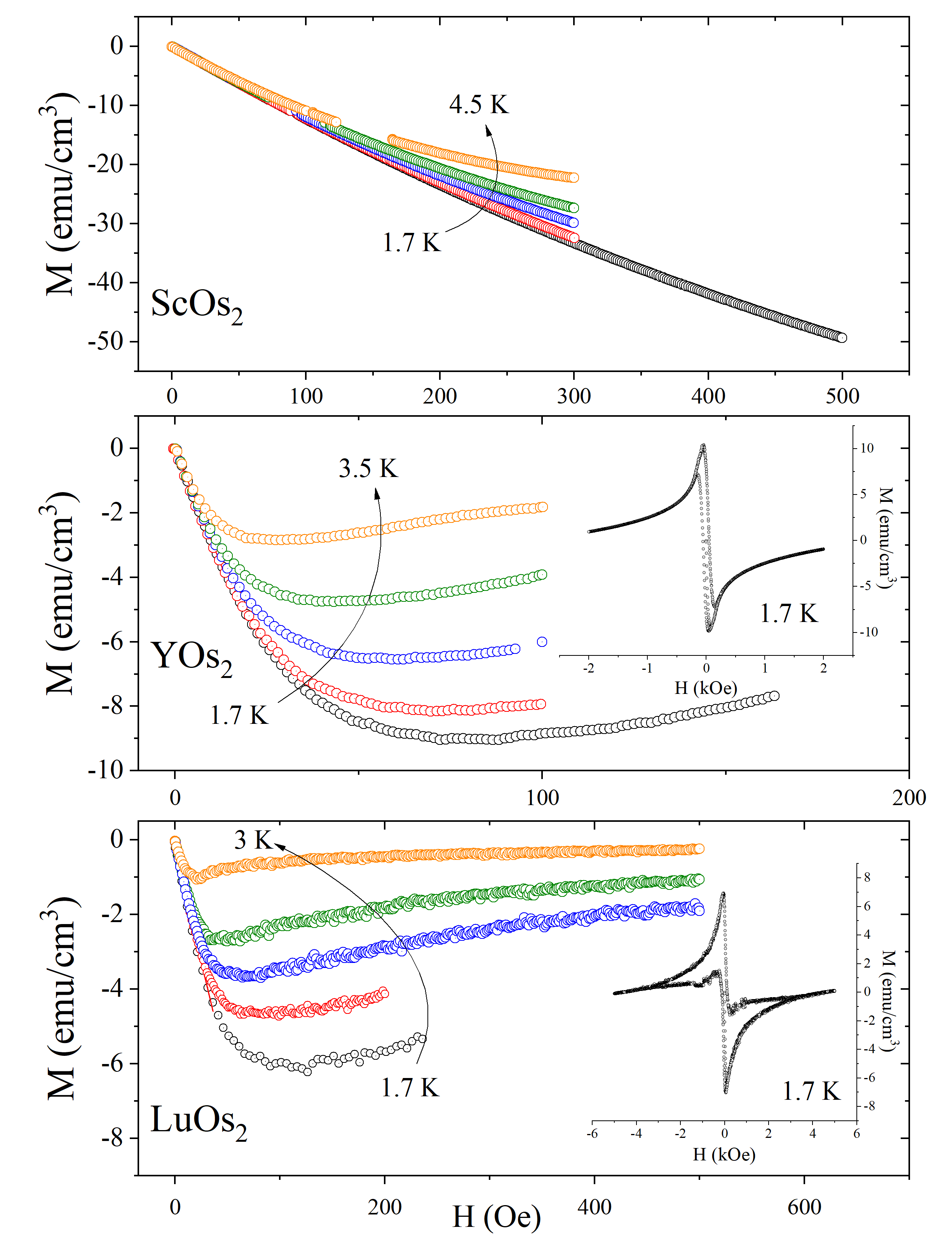


**Figure S3**. The field-dependent magnetization curves M(H) for ScOs_2_, YOs_2_, and LuOs_2_ taken at different temperatures.

**Electrical resistivity measurements**

For a single-band type-II BCS superconductor, the orbital upper critical field at 0 K can be estimated from the Werthamer–Helfand–Hohenberg expression^2,3^:

|  | $\mu_{0}H_{c2}\left( 0 \right)=-AT_{c}\left. \frac{d\mu_{0}H_{c2}}{dT} \right\vert_{T=T_{c}}$ | (1) |
| --- | --- | --- |

where A is the purity factor given by 0.693 for the dirty limit. **Table S1** presents the µ_0_H_c2_(0) value obtained from WHH and GL models for all investigated compounds.

**Table S1.** The upper critical field µ_0_H_c2_(0) estimated from WHH and GL models.

| ***μ_0_Hc_2_*(0)** | **Unit** | **ScOs_2_** | **YOs_2_** | **LuOs_2_** |
| --- | --- | --- | --- | --- |
| GL | T | 2.58(1) | 2.23(2) | 1.64(5) |
| WHH | T | 1.90 | 1.62 | 1.37 |
| µ_0_$H_{c2}^{p}\left( 0 \right)$ | T | 9.90 | 8.40 | 6.4 |

**References**

1. Prozorov, R. & Kogan, V. G. Effective Demagnetizing Factors of Diamagnetic Samples of Various Shapes. *Phys. Rev. Appl.* **10**, 014030 (2018).

2. Helfand, E. & Werthamer, N. R. Temperature and Purity Dependence of the Superconducting Critical Field, H c 2 . II. *Phys. Rev.* **147**, 288–294 (1966).

3. Werthamer, N. R., Helfand, E. & Hohenberg, P. C. Temperature and Purity Dependence of the Superconducting Critical Field, H c 2 . III. Electron Spin and Spin-Orbit Effects. *Phys. Rev.* **147**, 295–302 (1966).
